# Characteristics and outcomes of five patients with COVID‐19 and hematological malignancies

**DOI:** 10.1002/ccr3.4013

**Published:** 2021-06-10

**Authors:** Chiara Citterio, Antonio Lazzaro, Costanza Bosi, Maurice De Ponzio, Luigi Cavanna

**Affiliations:** ^1^ Oncology and Hematology Department Oncology Unit Piacenza General Hospital Piacenza Italy; ^2^ Division of Hematology and Bone Marrow Transplant Center Piacenza General Hospital Piacenza Italy; ^3^ Pharmacy Unit Piacenza General Hospital Piacenza Italy

**Keywords:** hematology, infectious diseases

## Abstract

Patients with hematological cancer are at major risk of developing infectious complication. The prevention and treatment of COVID‐19 in these patients is challenging. This experience, with the limitation of a small number of patients, highlights that early treatment of COVID‐19 can overcome the infection, also in hematological patients.

## Graphical abstract

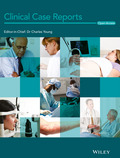

## 1 INTRODUCTION

We report five cases of Italian patients (two female, three male, age: 55, 63, 80, 70, and 79 years) with hematological malignancies and confirmed COVID‐19 pneumonia, managed with a multidisciplinary therapeutic approach that included antiviral therapy, hydroxychloroquine, antibiotic therapy, and management of comorbidities. Three patients were discharged in good clinical conditions; two died.

On March 2020, the World health Organization has characterized coronavirus infectious disease 2019 (COVID‐19) as a public health emergency of international concern and defined it a pandemic.^1^ In Italy, the most involved regions by COVID‐19 are Lombardy, Emilia Romagna, and Veneto. The city of Piacenza (Emilia Romagna region) is very near to the epicenter of the outbreak of COVID‐19, and the catastrophic nature of Lombardy's outbreak has been widely published.^2^ Recently, a report of the first 25 patients with solid tumor and COVID‐19 in a western country (Italy) was published.^3^ Hematological patients are known to have a greater sensitivity to infections, caused by the immunosuppression produced by treatments and closely related to their disease.^4^ Data on COVID‐19 in patients with hematological malignancies are fragmentary and poor.^5^, ^6^, ^7^ The current paper describes five patients with hematological cancer and COVID‐19 infection diagnosed and treated in the district of Piacenza (North Italy).

## 2 EXAMINATION

We retrospectively reviewed five patients with hematological malignancies and laboratory‐confirmed COVID‐19 infection, with reverse transcription‐polymerase chain reaction (RT‐PCR) in nasal‐pharyngeal swabs^8^; the COVID‐19 severity was classified as follow in accordance with the diagnosis and treatment of COVID‐19 guidelines^9^:

1. Mild type: The clinical symptoms are mild with no abnormal radiological findings.
2. Moderate type: Fever, cough, and other symptoms are presented with pneumonia on chest computed tomography or sonography.
3. Severe type: The disease is classified as if one of the following conditions is met:
  - Respiratory distress, respiratory rate ≥ 30 per men.
  - Oxygen saturation on room air at rest ≤ 93%.
  - Partial pressure of oxygen in arterial blood/fraction of inspired oxygen ≤ 300 mmHg.
4. Critical type: One of the following conditions has to be met:
  - Respiratory failure occurs, and mechanical ventilation is required.
  - Shock occurs.
  - Patients with other organ dysfunction need intensive care unit monitoring treatment.

Epidemiological features, physical examinations, laboratory assessment, and clinical outcome were described. Chest computerized tomography (CT) or X‐ray (RX) examinations were performed.^10^ These patients were hospitalized. This study was approved by the Local Ethics Committee. Each patient or legal representative signed an institutional informed consent in which he/she expressed his/her will to allow the use of clinical history data and the publication of these information for research purposes.

## 3 DIFFERENTIAL DIAGNOSIS, INVESTIGATIONS, AND TREATMENT

We report five cases of patients with hematological malignancies and confirmed COVID‐19 pneumonia, hospitalized between March and April 2020. All patients’ characteristics are reported in Table 1.

**TABLE 1:** Characteristic of subjects with hematological cancer and COVID‐19

	Case 01	Case 02	Case 03	Case 04	Case 05
Age	55	63	80	70	79
Sex	Female	Male	Male	Female	Male
Medical history	Arthrosis	Hypertension, hypercholesterolemia, atrial fibrillation, OSAS	COPD, prostate adenocarcinoma, chronic renal failure, gastrointestinal bleeding, ischemic injury duodenal ulcer, myelodysplastic syndrome	Rheumatoid arthritis, fibromyalgia, previous pancreatitis	HCV
Hematological disease	Acute myeloid leukemia	Gastric MALT NHL	Multiple Myeloma	Multiple Myeloma	Multiple Myeloma
Stage	NA	NA	III	III	NED
Hematological therapy	gilteritinib	rituximab + cyclophosphamide	Supportive therapy	bortezomib + radiotherapy	Follow up
Laboratory on admission	Thrombocytopenia, increased CRP, increased LDH, D‐dimer elevations	Anemia, increased CRP, increased LDH, D‐dimer elevations	Anemia, increased CRP, increased creatinine, hyperuricemia, increased LDH	Thrombocytopenia, increased CRP, increased LDH	increased CRP, increased LDH
Symptoms on admission	Fever	Fever, dyspnea	Vomit, abdominal pain	Fever, dyspnea	Fever, cough, diarrhea, dyspnea, respiratory failure
COVID‐19 severity	Severe	Severe	Severe	Severe	Severe
Duration of symptoms before treatment (days)	3	2	7	8	2
Chest CT scan at admission	Bilateral interstitial pneumonia	bilateral interstitial pneumonia	Negative^a^	Bilateral interstitial pneumonia	Bilateral interstitial pneumonia
Antibiotic therapy	teicoplanin + cefepime	azithromycin	linezolid	ceftriaxone + azithromycin	ceftriaxone + azithromycin
Antiviral therapy	darunavir + ritonavir	No	darunavir + cobicistat	lopinavir + ritonavir	darunavir + cobicistat
Hydroxychloroquine	Yes	Yes	Yes	Yes	Yes
Oxygen therapy	No	Yes	Yes	Yes	Yes
Anticoagulant therapy	Yes (enoxaparin 4000 UI/d)	Yes (enoxaparin 8000 UI/d)	No	No	Yes (enoxaparin 4000 UI/d)
Adverse event during hospitalization	Anemia	Anemia	Respiratory failure, fever, sepsis	Severe respiratory failure	Dysuria
Blood transfusion	Yes	Yes	Yes	No	No
Outcome	Discharged	Discharged	Death	Death	Discharged

Abbreviations: COPD, chronic obstructive pulmonary disease; CRP, C‐reactive protein; CT, computerized tomography; HCV, hepatitis C virus; MALT, mucosa‐associated lymphoid tissue; NA, not applicable; NED, no evidence of disease; NHL, non‐Hodgkin lymphoma; OSAS, obstructive sleep apnea syndrome.

^a^ CT scan performed 5 d after admission demonstrated bilateral pneumonia.

The mean age of the patients was 69.4 years (range 55‐80), three were male and two female. Three patients had multiple myeloma (MM), one acute myeloid leukemia, and one gastric B‐cell lymphoma. Three patients were in active hematological treatment, one in follow‐up, and one in supportive therapy. All patients had comorbidities, as shown in Table 1. At the time of admission, all patients showed an increase of CRP and LDH, two patients had anemia, two thrombocytopenia, and two an increased level of D‐dimer (Table 2). The immunoglobulin levels were tested in all the five patients, and it was low for everyone. All patients were tested for HIV and were negative. Fever and dyspnea were the most common symptoms at the admission; patients did not report anosmia and ageusia. Typical findings of chest CT images were observed in four patients at hospital admission, and in one patient, chest CT scan was initially negative, but 5 days later it demonstrated bilateral pneumonia. All the patients were treated with antibiotic therapy, three with anticoagulant therapy (enoxaparin), and one patient started treatment with linezolid for evidence of sepsis. Oxygen therapy was required in four patients. Four patients received antiviral treatment, including darunavir/cobicistat (n = 2), darunavir/ritonavir (n = 1), and lopinavir/ritonavir (n = 1). All the patients received hydroxychloroquine. Three patients received blood transfusion during the hospitalization for anemia, and three patients experienced respiratory failure. Three of them, treated with antiviral therapy and antibiotic, had gradually recovered and were discharged from the hospital. Two patients died for COVID‐19 pneumonia.

**TABLE 2:** Patient's laboratory results

	Normal range	Case 01	Case 02	Case 03	Case 04	Case 05
Date		19 Mar 2020	09 Apr 2020	11 Mar 2020	09 Mar 2020	28 Mar 2020
WBC count, ×10^3^/µL	4.00‐10.00	**3.56**	**10.83**	**148.13**	**3.19**	7.88
Neutrophil count, ×10^3^/µL	2.00‐8.00	**1.42**	**10.10**	**40.14**	3.08	6.92
Lymphocyte count, ×10^3^/µL	1.50‐4.00	1.74	**0.25**	6.07	**0.03**	**0.61**
Hemoglobin, gr/dL	12.0‐16.0	**9.2**	**8.5**	**9.4**	**10.5**	13
Platelet count, ×10^3^/µL	150‐450	**23**	**124**	**149**	**38**	344
Total bilirubin, mg/dL	0.00‐1.10	0.97	**1.20**	0.80	0.81	0.60
Creatinine, mg/dL	0.60‐1.00	0.62	0.89	**4.6**	0.54	0.81
Creatine kinase, U/L	0‐149	34	**577**	21	**325**	**223**
Prothrombin time, s	8‐14	15.5	**25.8**	13	**14.8**	13.8
APTT, s	26.5‐37.5	30.5	32.9	**26**	35.9	26.9
Fibrinogen, mg/dL	150‐400	**405**	**462**	NA	356	**619**
D‐dimer, ng/mL EFU	≤500	**4823**	**6359**	NA	NA	NA
CRP, mg/dL	0‐0.5	**5.03**	**17.17**	**16.20**	**12.36**	**17.98**
LDH, U/L	0‐247	**318**	**498**	**1184**	**557**	**425**

Note

Abnormal values are shown in bold.

Abbreviations: CRP, C‐reactive protein; EFU, equivalent fibrinogen units; LDH, lactate dehydrogenase; NA, not applicable.

## 4 DISCUSSION

There are few data about the risk of developing COVID‐19 in patients with hematological cancers. It is proven that patients with cancers involving the immune system or treated with immunochemotherapy regimen or immunomodulatory drugs, which suppress bone marrow function, are at risk of infections.^10^ Low immunoglobulin levels measured in these patients may have contributed to COVID‐19 susceptibility. In our case, patients presented with common symptoms of COVID‐19, but many patients present with atypical symptoms and some are very difficult to diagnose^11^ and require special attention. Studies about patients with COVID‐19, evidence variable hematological findings, such as lymphopenia, elevated LDH levels or altered coagulation tests, such as the prothrombin time (PT) and activated partial thromboplastin time (aPTT).^12^ Jin and colleagues^5^ reported COVID‐19 in a patient with chronic lymphocytic leukemia; they emphasized the fact that clinical and biochemical data of COVID‐19 might be partly masked by coexisting hematological disease. In this case, all patients presented increased CRP and LDH levels; other anomalies have been highlighted such as thrombocytopenia and anemia but they may be related to therapy of the hematological cancers rather than COVID‐19; indeed, case 05, without evidence of active hematological disease and in follow‐up, did not present abnormalities of the blood count. Zhou and colleagues^13^ evidence that, in COVID‐19 patients, D‐dimer level higher than 1 mg/mL was an independent risk factor of mortality and our patients 01 and 02 showed increased D‐dimer levels, LDH and CRP (Table 2) over the course of illness; however, they recovered and were discharged (Table 1).

Evidence of abnormal coagulation parameters associated with COVID‐19 appeared in early reports from China. Baseline characteristics of the first 99 patients hospitalized in Wuhan found that 36% had elevated D‐dimer and increased biomarkers of inflammation including CRP.^14^, ^15^ Different studies^16^, ^17^ report high proportion of aberrant coagulation in severe and critical patients with COVID‐19, they exhibited a hypercoagulable state, with elevated levels of D‐dimer, prolonged prothrombin time, and increased level of fibrinogen; caused by several factors like inflammatory status and aggressive immune response, which leads some patients to develop disseminated intravascular coagulation (DIC). Our data are consistent with data reported by Asakura and colleagues^18^; they evaluated the pathophysiology of DIC associated with COVID‐19 and observed an increased level of D‐dimer and fibrinogen related to a patient in hypercoagulable state. Tang and colleagues^19^ reported that 71.4% of nonsurvivors and 0.6% of survivors of COVID‐19 showed evidence of DIC. In this case reports, all patients treated with anticoagulant therapy had signs and symptoms improvement, in agreement with He and colleagues^7^ that early anticoagulation and early thrombolytic agents may provide better prognosis in COVID‐19 patients and Singhania and colleagues that recommended prophylactic anticoagulation in all patients with COVID‐19 unless contraindicated.^20^ Zhou et al^13^ reported that COVID‐19 patients, with older age and preexisting conditions (diabetes, hypertension, etc), have an increased risk of death. All the cases in this report had comorbidities, for which they received treatment even during hospitalization. Currently, no specific treatment is effective for treating COVID‐19, but HCQ with or without antiviral treatment has been incorporated in regional guideline to treat COVID‐19 and was performed in our patients.^21^, ^22^, ^23^ Zhan and colleagues^6^ reported the first case of a patient with MM, diagnosed with COVID‐19, treated with tocilizumab with resolution of the infectious disease. They observed that patients with MM are at high risk for COVID‐19 and treatment difficulties and evidence the need to determine the safety and efficacy of tocilizumab with randomized controlled trial. Two of the five patients of this series died, and they were affected by MM.

There are currently no data from rigorously conducted clinical trials evaluating COVID‐19 infection in hematological patients. He and colleagues^7^ evidence that hospitalized persons with hematological cancers have a similar case rate of COVID‐19 compared with normal healthcare providers but have more severe disease and a higher case fatality rate; in their case series, it seems related predominately to bacterial coinfections; also, in case 03 the patient developed sepsis from gram + cluster bacteria which worsened the patient's condition leading to the fatal outcome.

Every country is looking for a strategy to manage patients with hematological malignancies that reduce the risk of COVID‐19 for patients and optimizes the use of resources, in Seattle (United States) specialists who manage adult patients with hematological malignancies, lay out treatment guidelines with treatment recommendations for different hematological malignancies^24^; some of the recommended strategies are using oral and outpatient regimens, increasing telemedicine visits, and avoiding or omitting therapies known to be associated with higher risk of viral infections.

Experience in managing COVID‐19 patients suggests that early screening, diagnosis, and treatment are critical to prevent complications and to improve clinical outcome; Lin and colleagues^25^ defined three phases of the infection: viremia phase, pneumonia phase, and, based on the course of the disease, a severe or recovery phase. The immune response depends on different factor: immune system, comorbidities, age, etc, and is concentrated in the first or second phase, but patients with immune dysfunction have a greater risk of aggravation and subsequent death, already in the first phase. There were several limitations to this study: The sample size is small, only five patients with hematological malignancies and COVID‐19 were reported, and subject had heterogeneous hematological diagnoses and disease stage. However, patients with hematological cancer can overcame COVID‐19 pneumonia when promptly diagnosed and precociously treated as demonstrated in 3 of 5 patients presented here. The three patients that overcame the infection were treated within 3 days from infection; conversely, the patients that died received treatment after 7 and 8 days, respectively, and these data underline the importance of early treatment.^26^

## CONFLICT OF INTEREST

The authors declare that they have no competing interests.

## AUTHOR CONTRIBUTIONS

CC: contributed to the conception and design of the work, acquisition, analysis, and interpretation of data for the work, drafting the work and revising it critically for important intellectual content, and final approval of the version to be published. LA: contributed to drafting the work and revising it critically for important intellectual content, and final approval of the version to be published. BC: contributed to drafting the work and revising it critically for important intellectual content, and final approval of the version to be published. DPM: contributed to drafting the work and revising it critically for important intellectual content, and final approval of the version to be published. CL: contributed to the conception and design of the work, acquisition, analysis, and interpretation of data for the work, drafting the work and revising it critically for important intellectual content, and final approval of the version to be published.

## ETHICS APPROVAL AND CONSENT TO PARTICIPATE

This study was approved by the Local Ethics Committee (institutional review board—IRB—approval number 494/2020/OSS*/AUSLPC). Each patient or legal representative signed an institutional informed consent in which he/she expressed his/her will to allow the use of clinical history data and the publication of these information for research purposes.

## Data Availability

The datasets generated and/or analyzed during the current study are not publicly available due to the Hospital privacy policies, but are available from the corresponding author upon reasonable request.
